# Padé approximant related to the Wallis formula

**DOI:** 10.1186/s13660-017-1406-z

**Published:** 2017-06-08

**Authors:** Long Lin, Wen-Cheng Ma, Chao-Ping Chen

**Affiliations:** 1 School of Mathematics and Informatics, Henan Polytechnic University, Jiaozuo City, Henan Province 454000 China; 2 College of Chemistry and Chemical Engineering, Henan Polytechnic University, Jiaozuo City, Henan Province 454000 China

**Keywords:** 33B15, 26D07, 41A60, gamma function, psi function, Wallis ratio, inequality, approximation

## Abstract

Based on the Padé approximation method, in this paper we determine the coefficients $a_{j}$ and $b_{j}$ such that

$$ \pi= \biggl(\frac{(2n)!!}{(2n-1)!!} \biggr)^{2} \biggl\{ \frac {n^{k}+a_{1}n^{k-1}+\cdots+a_{k}}{n^{k+1}+b_{1}n^{k}+\cdots+b_{k+1}}+O \biggl(\frac{1}{n^{2k+3}} \biggr) \biggr\} ,\quad n\to\infty, $$

where $k\geq0$ is any given integer. Based on the obtained result, we establish a more accurate formula for approximating *π*, which refines some known results.

## Introduction

It is well known that the number *π* satisfies the following inequalities:

1.1 $$ \begin{aligned} \frac{2}{2n+1} \biggl( \frac{(2n)!!}{(2n-1)!!} \biggr)^{2}< \pi< \frac {1}{n} \biggl( \frac{(2n)!!}{(2n-1)!!} \biggr)^{2}, \quad n \in \mathbb{N}:=\{1,2,3,\ldots\}, \end{aligned} $$

where

$$\begin{aligned} (2n)!!=2\cdot4\cdot6\cdots(2n)=2^{n} n!,\qquad (2n-1)!!=1\cdot3\cdot 5 \cdots(2n-1). \end{aligned}$$

This result is due to Wallis (see [1]).

Based on a basic theorem in mathematical statistics concerning unbiased estimators with minimum variance, Gurland [1] yielded a closer approximation to *π* than that afforded by (1.1), namely,

1.2 $$ \begin{aligned} \frac{4n+3}{(2n+1)^{2}} \biggl( \frac{(2n)!!}{(2n-1)!!} \biggr)^{2}< \pi< \frac {4}{4n+1} \biggl( \frac{(2n)!!}{(2n-1)!!} \biggr)^{2}, \quad n \in \mathbb{N}. \end{aligned} $$

By using (1.2), Brutman [2] and Falaleev [3] established estimates of the Landau constants.

Mortici [4], Theorem 2, improved Gurland’s result (1.2) and obtained the following double inequality:

1.3 $$\begin{aligned} & \biggl(\frac{n+\frac{1}{4}}{n^{2}+\frac{1}{2}n+\frac{3}{32}}+\frac{9}{ 2{,}048n^{5}}-\frac{45}{ 8{,}192n^{6}} \biggr) \biggl(\frac{(2n)!!}{(2n-1)!!} \biggr)^{2} \\ &\quad < \pi< \biggl(\frac{n+\frac{1}{4}}{n^{2}+\frac{1}{2}n+\frac{3}{32}}+\frac{9}{ 2{,}048n^{5}} \biggr) \biggl( \frac{(2n)!!}{(2n-1)!!} \biggr)^{2},\quad n \in \mathbb{N}. \end{aligned}$$

We see from (1.3) that

1.4 $$ \pi= \biggl(\frac{(2n)!!}{(2n-1)!!} \biggr)^{2} \biggl\{ \frac{n+\frac {1}{4}}{n^{2}+\frac{1}{2}n+\frac{3}{32}}+O \biggl(\frac{1}{n^{5}} \biggr) \biggr\} ,\quad n\to\infty. $$

Based on the Padé approximation method, in this paper we develop the approximation formula (1.4) to produce a general result. More precisely, we determine the coefficients $a_{j}$ and $b_{j}$ such that

1.5 $$ \pi= \biggl(\frac{(2n)!!}{(2n-1)!!} \biggr)^{2} \biggl\{ \frac {n^{k}+a_{1}n^{k-1}+\cdots+a_{k}}{n^{k+1}+b_{1}n^{k}+\cdots+b_{k+1}}+O \biggl(\frac{1}{n^{2k+3}} \biggr) \biggr\} ,\quad n\to\infty, $$

where $k\geq0$ is any given integer. Based on the obtained result, we establish a more accurate formula for approximating *π*, which refines some known results.

The numerical values given in this paper have been calculated via the computer program MAPLE 13.

## Lemmas

Euler’s gamma function $\Gamma(x)$ is one of the most important functions in mathematical analysis and has applications in diverse areas. The logarithmic derivative of $\Gamma(x)$, denoted by $\psi(x)=\Gamma'(x)/\Gamma(x)$, is called the psi (or digamma) function.

The following lemmas are required in the sequel.

### Lemma 2.1

[5]

*Let* $r\neq0$ *be a given real number and* $\ell\geq0$ *be a given integer*. *The following asymptotic expansion holds*:

2.1 $$\begin{aligned} \frac{\Gamma(x+1)}{\Gamma (x+\frac{1}{2} )}&\sim\sqrt{x} \Biggl(1+\sum _{j=1}^{\infty}\frac{p_{j}}{x^{j}} \Biggr)^{x^{\ell}/r},\quad x\to\infty, \end{aligned}$$

*with the coefficients* $p_{j}\equiv p_{j}(\ell,r)\ (j\in\mathbb{N})$ *given by*

2.2 $$\begin{aligned} p_{j}=\sum\frac{r^{k_{1}+k_{2}+\cdots+k_{j}}}{k_{1}!k_{2}!\cdots k_{j}!} \biggl( \frac{(2^{2}-1)B_{2}}{1\cdot1\cdot2^{2}} \biggr)^{k_{1}} \biggl(\frac{(2^{4}-1)B_{4}}{2\cdot3\cdot2^{4}} \biggr)^{k_{2}}\cdots \biggl(\frac {(2^{2j}-1)B_{2j}}{j(2j-1)2^{2j}} \biggr)^{k_{j}}, \end{aligned}$$

*where* $B_{j}$ *are the Bernoulli numbers summed over all nonnegative integers* $k_{j}$ *satisfying the equation*

$$\begin{aligned} (1+\ell)k_{1}+(3+\ell)k_{2}+\cdots+(2j+ \ell-1)k_{j}=j. \end{aligned}$$

In particular, setting $(\ell, r)=(0, -2)$ in (2.1) yields

2.3 $$\begin{aligned} x \biggl(\frac{\Gamma (x+\frac{1}{2} )}{\Gamma(x+1)} \biggr)^{2}\sim 1+\sum _{j=1}^{\infty}\frac{c_{j}}{x^{j}}, \quad x\to\infty, \end{aligned}$$

where the coefficients $c_{j}\equiv p_{j}(0, -2)\ (j\in\mathbb{N})$ are given by

2.4 $$\begin{aligned} c_{j}=\sum\frac{(-2)^{k_{1}+k_{2}+\cdots+k_{j}}}{k_{1}!k_{2}!\cdots k_{j}!} \biggl( \frac{(2^{2}-1)B_{2}}{1\cdot1\cdot2^{2}} \biggr)^{k_{1}} \biggl(\frac{(2^{4}-1)B_{4}}{2\cdot3\cdot2^{4}} \biggr)^{k_{2}}\cdots \biggl(\frac {(2^{2j}-1)B_{2j}}{j(2j-1)2^{2j}} \biggr)^{k_{j}}, \end{aligned}$$

summed over all nonnegative integers $k_{j}$ satisfying the equation

$$\begin{aligned} k_{1}+3k_{2}+\cdots+(2j-1)k_{j}=j. \end{aligned}$$

### Lemma 2.2

[5]

*Let* $m, n\in\mathbb{N}$. *Then*, *for* $x>0$,

2.5 $$\begin{aligned} \sum_{j=1}^{2m} \biggl(1- \frac{1}{2^{2j}} \biggr)\frac{2B_{2j}}{(2j)!}\frac {(2j+n-2)!}{x^{2j+n-1}}&< (-1)^{n} \biggl(\psi^{(n-1)}(x+1)-\psi ^{(n-1)} \biggl(x+\frac{1}{2} \biggr) \biggr)+\frac{(n-1)!}{2x^{n}} \\ & < \sum_{j=1}^{2m-1} \biggl(1- \frac{1}{2^{2j}} \biggr)\frac {2B_{2j}}{(2j)!}\frac{(2j+n-2)!}{x^{2j+n-1}}. \end{aligned}$$

In particular, we have

2.6 $$\begin{aligned} U(x)< \psi(x+1)-\psi \biggl(x+\frac{1}{2} \biggr)< V(x), \end{aligned}$$

where

$$\begin{aligned} V(x)={}&\frac{1}{2x}-\frac{1}{8x^{2}}+\frac{1}{64x^{4}}- \frac{1}{128x^{6}}+\frac {17}{2{,}048x^{8}}-\frac{31}{2{,}048x^{10}}+\frac{691}{16{,}384x^{12}}\\ &{}- \frac {5{,}461}{32{,}768x^{14}}+\frac{929{,}569}{1{,}048{,}576x^{16}} \end{aligned}$$

and

$$\begin{aligned} U(x)=V(x)-\frac{3{,}202{,}291}{524{,}288x^{18}}. \end{aligned}$$

For our later use, we introduce Padé approximant (see [6–11]). Let *f* be a formal power series

2.7 $$\begin{aligned} f(t)=c_{0}+c_{1}t+c_{2}t^{2}+ \cdots. \end{aligned}$$

The Padé approximation of order $(p, q)$ of the function *f* is the rational function, denoted by

2.8 $$\begin{aligned}{} [p/q]_{f}(t)=\frac{\sum_{j=0}^{p}a_{j}t^{j}}{1+\sum_{j=1}^{q}b_{j}t^{j}}, \end{aligned}$$

where $p\geq0$ and $q\geq1$ are two given integers, the coefficients $a_{j}$ and $b_{j}$ are given by (see [6–8, 10, 11])

2.9 $$\begin{aligned} \textstyle\begin{cases} a_{0}=c_{0},\\ a_{1}=c_{0}b_{1}+c_{1},\\ a_{2}=c_{0}b_{2}+c_{1}b_{1}+c_{2},\\ \vdots\\ a_{p} = c_{0}b_{p}+\cdots+ c_{p-1}b_{1} + c_{p},\\ 0 = c_{p+1} + c_{p}b_{1} + \cdots+ c_{p-q+1}b_{q},\\ \vdots\\ 0 = c_{p+q} + c_{p+q-1}b_{1} + \cdots+ c_{p}b_{q}, \end{cases}\displaystyle \end{aligned}$$

and the following holds:

2.10 $$\begin{aligned}{} [p/q]_{f}(t)- f (t) = O\bigl(t^{p+q+1} \bigr). \end{aligned}$$

Thus, the first $p + q + 1$ coefficients of the series expansion of $[p/q]_{f}$ are identical to those of *f*. Moreover, we have (see [9])

2.11 $$ \begin{aligned} &[p/q]_{f}(t)= \frac{\left \vert {\scriptsize\begin{matrix}{} t^{q}f_{p-q}(t) & t^{q-1}f_{p-q+1}(t) &\cdots &f_{p}(t) \cr c_{p-q+1} & c_{p-q+2} &\cdots &c_{p+1} \cr \vdots &\vdots &\ddots &\vdots \cr c_{p} & c_{p+1} &\cdots &c_{p+q} \end{matrix}} \right \vert }{ \left \vert {\scriptsize\begin{matrix}{} t^{q} & t^{q-1} &\cdots &1 \cr c_{p-q+1} & c_{p-q+2} &\cdots &c_{p+1} \cr \vdots &\vdots &\ddots &\vdots \cr c_{p} & c_{p+1} &\cdots &c_{p+q} \end{matrix}} \right \vert }, \end{aligned} $$

with $f_{n}(x) = c_{0}+ c_{1}x+ \cdots+ c_{n}x^{n}$, the *n*th partial sum of the series *f* in (2.7).

## Main results

Let

3.1 $$\begin{aligned} f(x)=x \biggl(\frac{\Gamma (x+\frac{1}{2} )}{\Gamma(x+1)} \biggr)^{2}. \end{aligned}$$

It follows from (2.3) that, as $x\to \infty$,

3.2 $$\begin{aligned} f(x)\sim\sum_{j=0}^{\infty} \frac{c_{j}}{x^{j}}={}&1-\frac{1}{4x}+\frac {1}{32x^{2}}+\frac{1}{128x^{3}}- \frac{5}{2{,}048x^{4}}-\frac{23}{8{,}192x^{5}}+\frac {53}{65{,}536x^{6}} \\ &{}+\frac{593}{262{,}144x^{7}}- \cdots, \end{aligned}$$

with the coefficients $c_{j}$ given by (2.4). In what follows, the function *f* is given in (3.1).

Based on the Padé approximation method, we now give a derivation of formula (1.4). To this end, we consider

$$\begin{aligned}{} [1/2]_{f}(x)=\frac{\sum_{j=0}^{1}a_{j}x^{-j}}{1+\sum_{j=1}^{2}b_{j}x^{-j}}. \end{aligned}$$

Noting that

$$\begin{aligned} c_{0}=1,\qquad c_{1}=-\frac{1}{4}, \qquad c_{2}= \frac{1}{32}, \qquad c_{3}=\frac{1}{128} \end{aligned}$$

holds, we have, by (2.9),

$$\begin{aligned} \textstyle\begin{cases} a_{0}=1,\\ a_{1}=b_{1}-\frac{1}{4},\\ 0 =\frac{1}{32}- \frac{1}{4}b_{1}+b_{2}, \\ 0 = \frac{1}{128} + \frac{1}{32}b_{1}-\frac{1}{4}b_{2}, \end{cases}\displaystyle \end{aligned}$$

that is,

$$\begin{aligned} a_{0}=1,\qquad a_{1}=\frac{1}{4},\qquad b_{1}= \frac{1}{2},\qquad b_{2} = \frac{3}{32}. \end{aligned}$$

We thus obtain that

3.3 $$ [1/2]_{f}(x)= \frac{1+\frac{1}{4x}}{1+\frac{1}{2x}+\frac{3}{32x^{2}}}, $$

and we have, by (2.10),

3.4 $$ x \biggl(\frac{\Gamma (x+\frac{1}{2} )}{\Gamma(x+1)} \biggr)^{2}- \frac{1+\frac{1}{4x}}{1+\frac{1}{2x}+\frac{3}{32x^{2}}}= O \biggl(\frac {1}{x^{4}} \biggr), \quad x\to\infty. $$

Noting that

3.5 $$ \frac{\Gamma(n+\frac{1}{2})}{\Gamma(n+1)}=\sqrt{\pi}\cdot\frac {(2n-1)!!}{(2n)!!}, \quad n\in \mathbb{N} \text{ (the Wallis ratio)} $$

holds, replacing *x* by *n* in (3.4) yields (1.4).

From the Padé approximation method introduced in Section 2 and the asymptotic expansion (3.2), we obtain a general result given by Theorem 3.1. As a consequence, we obtain (1.5).

### Theorem 3.1

*The Padé approximation of order* $(p, q)$ *of the asymptotic formula of the function* $f(x)=x (\frac{\Gamma (x+\frac{1}{2} )}{\Gamma (x+1)} )^{2}$ (*at the point* $x=\infty$) *is the following rational function*:

3.6 $$\begin{aligned}{} [p/q]_{f}(x)=\frac{1+\sum_{j=1}^{p}a_{j}x^{-j}}{1+\sum_{j=1}^{q}b_{j}x^{-j}}=x \biggl( \frac{x^{p}+a_{1}x^{p-1}+\cdots +a_{p}}{x^{q}+b_{1}x^{q-1}+\cdots+b_{q}} \biggr), \end{aligned}$$

*where* $p\geq0$ *and* $q\geq1$ *are two given integers and* $q=p+1$ (*an empty sum is understood to be zero*), *the coefficients* $a_{j}$ *and* $b_{j}$ *are given by*

3.7 $$\begin{aligned} \textstyle\begin{cases} a_{1}=b_{1}+c_{1},\\ a_{2}=b_{2}+c_{1}b_{1}+c_{2},\\ \vdots\\ a_{p} = b_{p}+\cdots+ c_{p-1}b_{1} + c_{p},\\ 0 = c_{p+1} + c_{p}b_{1} + \cdots+ c_{p-q+1}b_{q},\\ \vdots\\ 0 = c_{p+q} + c_{p+q-1}b_{1} + \cdots+ c_{p}b_{q}, \end{cases}\displaystyle \end{aligned}$$

*and* $c_{j}$ *is given in* (2.4), *and the following holds*:

3.8 $$\begin{aligned} f (x) -[p/q]_{f}(x) = O \biggl(\frac{1}{x^{p+q+1}} \biggr),\quad x\to\infty. \end{aligned}$$

*Moreover*, *we have*

3.9 $$ \begin{aligned} &[p/q]_{f}(x)= \frac{\left \vert {\scriptsize\begin{matrix}{} \frac{1}{x^{q}}f_{p-q}(x) & \frac{1}{x^{q-1}}f_{p-q+1}(t) &\cdots &f_{p}(t) \cr c_{p-q+1} & c_{p-q+2} &\cdots &c_{p+1} \cr \vdots &\vdots &\ddots &\vdots \cr c_{p} & c_{p+1} &\cdots &c_{p+q} \end{matrix}} \right \vert }{ \left \vert {\scriptsize\begin{matrix}{} \frac{1}{x^{q}} & \frac{1}{x^{q-1}} &\cdots &1 \cr c_{p-q+1} & c_{p-q+2} &\cdots &c_{p+1} \cr \vdots &\vdots &\ddots &\vdots \cr c_{p} & c_{p+1} &\cdots &c_{p+q} \end{matrix}} \right \vert }, \end{aligned} $$

*with* $f_{n}(x)=\sum_{j=0}^{n}\frac{c_{j}}{x^{j}}$, *the* *nth partial sum of the asymptotic series* (3.2).

### Remark 3.1

Using (3.9), we can also derive (3.3). Indeed, we have

$$ \begin{aligned}{} [1/2]_{f}(x)&=\frac{\left \vert {\scriptsize\begin{matrix}{} \frac{1}{x^{2}}f_{-1}(x) & \frac{1}{x}f_{0}(x) &f_{1}(x) \cr c_{0} &c_{1} &c_{2} \cr c_{1} &c_{2} &c_{3} \end{matrix}} \right \vert }{ \left \vert {\scriptsize\begin{matrix}{} \frac{1}{x^{2}} &\frac{1}{x} &1 \cr c_{0} &c_{1} &c_{2} \cr c_{1} &c_{2} &c_{3} \end{matrix}} \right \vert } = \frac{\left \vert {\scriptsize\begin{matrix}{} 0 & \frac{1}{x} &1-\frac{1}{4x}\cr 1 &-\frac{1}{4} &\frac{1}{32} \cr -\frac{1}{4} &\frac{1}{32} &\frac{1}{128} \end{matrix}} \right \vert }{ \left \vert {\scriptsize\begin{matrix}{} \frac{1}{x^{2}} &\frac{1}{x} &1 \cr 1 &-\frac{1}{4} &\frac{1}{32} \cr -\frac{1}{4} &\frac{1}{32} &\frac{1}{128} \end{matrix}} \right \vert }=\frac{1+\frac{1}{4x}}{1+\frac{1}{2x}+\frac{3}{32x^{2}}}. \end{aligned} $$

Replacing *x* by *n* in (3.8) applying (3.5), we obtain the following corollary.

### Corollary 3.1

*As* $n\to\infty$,

3.10 $$ \pi= \biggl(\frac{(2n)!!}{(2n-1)!!} \biggr)^{2} \biggl\{ \frac{n^{p}+\sum_{j=1}^{p}a_{j}n^{p-j}}{n^{q}+\sum_{j=1}^{q}b_{j}n^{q-j}}+O \biggl(\frac {1}{n^{p+q+2}} \biggr) \biggr\} ,\quad n\to\infty, $$

*where* $p\geq0$ *and* $q\geq1$ *are two given integers and* $q=p+1$, *and the coefficients* $a_{j}$ *and* $b_{j}$ *are given by* (3.7).

### Remark 3.2

Setting $(p, q)=(k, k+1)$ in (3.10) yields (1.5).

Setting

$$ (p, q)=(4, 5) \quad\text{and}\quad (p, q)=(5, 6) $$

in (3.10), respectively, we find

3.11 $$ \pi= \biggl(\frac{(2n)!!}{(2n-1)!!} \biggr)^{2} \biggl\{ \frac{n^{4}+n^{3}+\frac {107}{64}n^{2}+\frac{91}{128}n+\frac{789}{4{,}096}}{n^{5}+\frac{5}{4}n^{4}+\frac {125}{64}n^{3}+\frac{295}{256}n^{2}+\frac{1{,}689}{4{,}096}n+\frac {945}{16{,}384}}+O \biggl(\frac{1}{n^{11}} \biggr) \biggr\} $$

and

3.12 $$\begin{aligned} \pi={}& \biggl(\frac{(2n)!!}{(2n-1)!!} \biggr)^{2} \\ &{}\times \biggl\{ \frac{n^{5}+\frac {5}{4}n^{4}+\frac{51}{16}n^{3}+\frac{133}{64}n^{2}+\frac{5{,}243}{4{,}096}n+\frac {3{,}867}{16{,}384}}{n^{6}+\frac{3}{2}n^{5}+\frac{113}{32}n^{4}+\frac {93}{32}n^{3}+\frac{7{,}729}{4{,}096}n^{2}+\frac{4{,}881}{8{,}192}n+\frac {10{,}395}{131{,}072}}+O \biggl(\frac{1}{n^{13}} \biggr) \biggr\} \end{aligned}$$

as $n\to\infty$.

Formulas (3.11) and (3.12) motivate us to establish the following theorem.

### Theorem 3.2

*The following inequality holds*:

3.13 $$\begin{aligned} &\frac{x^{5}+\frac{5}{4}x^{4}+\frac{51}{16}x^{3}+\frac{133}{64}x^{2}+\frac {5{,}243}{4{,}096}x+\frac{3{,}867}{16{,}384}}{x^{6}+\frac{3}{2}x^{5}+\frac {113}{32}x^{4}+\frac{93}{32}x^{3}+\frac{7{,}729}{4{,}096}x^{2}+\frac {4{,}881}{8{,}192}x+\frac{10{,}395}{131{,}072}} \\ &\quad < \biggl(\frac{\Gamma(x+\frac {1}{2})}{\Gamma(x+1)} \biggr)^{2} \\ &\quad < \frac{x^{4}+x^{3}+\frac{107}{64}x^{2}+\frac{91}{128}x+\frac {789}{4{,}096}}{x^{5}+\frac{5}{4}x^{4}+\frac{125}{64}x^{3}+\frac {295}{256}x^{2}+\frac{1{,}689}{4{,}096}x+\frac{945}{16{,}384}}. \end{aligned}$$

*The left*-*hand side inequality holds for* $x\geq4$, *while the right*-*hand side inequality is valid for* $x\geq3$.

### Proof

It suffices to show that

$$\begin{aligned} F(x)>0 \quad\text{for } x\geq4 \quad\text{and}\quad G(x)< 0 \quad\text{for } x\geq3, \end{aligned}$$

where

$$\begin{aligned} F(x)=2\ln \biggl(\frac{\Gamma(x+\frac{1}{2})}{\Gamma(x+1)} \biggr)-\ln\frac {x^{5}+\frac{5}{4}x^{4}+\frac{51}{16}x^{3}+\frac{133}{64}x^{2}+\frac {5{,}243}{4{,}096}x+\frac{3{,}867}{16{,}384}}{x^{6}+\frac{3}{2}x^{5}+\frac {113}{32}x^{4}+\frac{93}{32}x^{3}+\frac{7{,}729}{4{,}096}x^{2}+\frac {4{,}881}{8{,}192}x+\frac{10{,}395}{131{,}072}} \end{aligned}$$

and

$$\begin{aligned} G(x)=2\ln \biggl(\frac{\Gamma(x+\frac{1}{2})}{\Gamma(x+1)} \biggr)-\ln\frac {x^{4}+x^{3}+\frac{107}{64}x^{2}+\frac{91}{128}x+\frac{789}{4{,}096}}{x^{5}+\frac {5}{4}x^{4}+\frac{125}{64}x^{3}+\frac{295}{256}x^{2}+\frac{1{,}689}{4{,}096}x+\frac {945}{16{,}384}}. \end{aligned}$$

Using the following asymptotic expansion (see [12]):

3.14 $$\begin{aligned} \biggl[\frac{\Gamma(x+\frac{1}{2})}{\Gamma(x+1)} \biggr]^{2}\sim{}& \frac{1}{x}\exp \biggl(-\frac{1}{4x}+\frac{1}{96x^{3}}- \frac {1}{320x^{5}}+\frac{17}{7{,}168x^{7}}-\frac{31}{9{,}216x^{9}} \\ &{} +\frac{691}{90{,}112x^{11}}-\frac{5{,}461}{212{,}992x^{13}}+\frac {929{,}569}{7{,}864{,}320x^{15}}-\cdots \biggr),\quad x\to \infty, \end{aligned}$$

we obtain that

$$\begin{aligned} \lim_{x\to\infty}F(x)=0 \quad\text{and}\quad \lim_{x\to\infty}G(x)=0. \end{aligned}$$

Differentiating $F(x)$ and applying the first inequality in (2.6), we find

$$\begin{aligned} F'(x)&=-2 \biggl[\psi(x+1)-\psi \biggl(x+\frac{1}{2} \biggr) \biggr]+\frac {P_{10}(x)}{P_{11}(x)} \\ &< -2U(x)+\frac{P_{10}(x)}{P_{11}(x)}=-\frac{P_{16}(x-4)}{524{,}288x^{18}P_{11}(x)}, \end{aligned}$$

where

$$\begin{aligned} P_{10}(x)={}&4\bigl(20{,}998{,}323+301{,}244{,}208x+ 1{,}329{,}622{,}624x^{2}+3{,}532{,}111{,}872x^{3}\\ &{}+6{,}831{,}390{,}720x^{4} +8{,}950{,}906{,}880x^{5}+9{,}510{,}060{,}032x^{6}\\ &{}+6{,}476{,}005{,}376x^{7}+4{,}244{,}635{,}648x^{8} +1{,}342{,}177{,}280x^{9}+536{,}870{,}912x^{10}\bigr), \\ P_{11}(x)={}&\bigl(16{,}384x^{5}+20{,}480x^{4}+52{,}224x^{3}+34{,}048x^{2}+20{,}972x+3{,}867 \bigr) \\ & {}\times\bigl(131{,}072x^{6}+196{,}608x^{5}+462{,}848x^{4}+380{,}928x^{3} +247{,}328x^{2}\\ &{}+78{,}096x+10{,}395 \bigr) \end{aligned}$$

and

$$\begin{aligned} P_{16}(x)={}&73{,}399{,}302{,}245{,}132{,}658{,}732{,}474+401{,}687{,}666{,}421{,}636{,}714{,}876{,}048x \\ &{} +882{,}663{,}824{,}965{,}187{,}436{,}960{,}169x^{2}\\ &{}+1{,}129{,}813{,}735{,}156{,}766{,}429{,}414{,}420x^{3} \\ &{} +975{,}385{,}167{,}000{,}268{,}446{,}720{,}384x^{4}\\ &{}+611{,}802{,}531{,}654{,}753{,}268{,}270{,}848x^{5} \\ & +290{,}696{,}674{,}545{,}996{,}984{,}221{,}376x^{6}\\ &{}+107{,}149{,}026{,}028{,}490{,}487{,}475{,}968x^{7} \\ &{} +31{,}018{,}031{,}026{,}615{,}120{,}693{,}760x^{8}\\ &{}+7{,}080{,}024{,}048{,}117{,}231{,}228{,}928x^{9} \\ &{} +1{,}270{,}066{,}473{,}244{,}063{,}756{,}800x^{10}+177{,}136{,}978{,}237{,}041{,}715{,}200x^{11} \\ & {}+18{,}824{,}726{,}793{,}935{,}462{,}400x^{12}+1{,}473{,}208{,}721{,}923{,}276{,}800x^{13} \\ &{} +80{,}051{,}720{,}723{,}251{,}200x^{14} +2{,}698{,}074{,}228{,}326{,}400x^{15}\\ &{}+42{,}489{,}357{,}926{,}400x^{16}. \end{aligned}$$

Hence, $F'(x)<0$ for $x\geq4$, and we have

$$\begin{aligned} F(x)>\lim_{t\to\infty}F(t)=0, \quad x\geq4. \end{aligned}$$

Differentiating $G(x)$ and applying the second inequality in (2.6), we find

$$\begin{aligned} G'(x)&=-2 \biggl[\psi(x+1)-\psi \biggl(x+\frac{1}{2} \biggr) \biggr]+\frac {4P_{8}(x)}{P_{9}(x)}>-2V(x)+\frac{4P_{8}(x)}{P_{9}(x)}\\ &=\frac {P_{14}(x-3)}{524{,}288x^{16}P_{9}(x)}, \end{aligned}$$

where

$$\begin{aligned} P_{8}(x)={}&16{,}777{,}216x^{8}+33{,}554{,}432x^{7}+72{,}351{,}744x^{6} +79{,}167{,}488x^{5}+75{,}583{,}488x^{4}\\ &{}+45{,}043{,}712x^{3} +18{,}211{,}328x^{2}+4{,}212{,}480x+644{,}661, \\ P_{9}(x)={}&\bigl(4{,}096x^{4}+4{,}096x^{3}+6{,}848x^{2}+2{,}912x+789 \bigr) \\ &{} \times\bigl(16{,}384x^{5}+20{,}480x^{4}+32{,}000x^{3}+18{,}880x^{2}+6{,}756x+945 \bigr) \end{aligned}$$

and

$$\begin{aligned} P_{14}(x)={}&427{,}884{,}340{,}806{,}856{,}575+ 5{,}508{,}337{,}280{,}234{,}438{,}700x\\ &{}+16{,}278{,}641{,}070{,}340{,}979{,}232x^{2} \\ &{} +25{,}110{,}186{,}749{,}213{,}013{,}376x^{3} +25{,}009{,}399{,}125{,}661{,}680{,}960x^{4}\\ &{}+17{,}642{,}792{,}222{,}808{,}253{,}696x^{5} \\ &{} +9{,}230{,}356{,}959{,}310{,}493{,}184x^{6} +3{,}661{,}094{,}552{,}739{,}530{,}752x^{7}\\ &{}+1{,}108{,}535{,}832{,}992{,}448{,}000x^{8} \\ &{} +255{,}024{,}028{,}762{,}675{,}200x^{9} +43{,}854{,}087{,}132{,}979{,}200x^{10}\\ &{}+5{,}462{,}018{,}666{,}496{,}000x^{11} \\ &{} +465{,}495{,}496{,}704{,}000x^{12}+24{,}287{,}993{,}856{,}000x^{13}\\ &{}+585{,}252{,}864{,}000x^{14}. \end{aligned}$$

Hence, $G'(x)>0$ for $x\geq3$, and we have

$$\begin{aligned} G(x)< \lim_{t\to\infty}G(t)=0,\quad x\geq3. \end{aligned}$$

The proof is complete. □

### Corollary 3.2

*For* $n \in \mathbb{N}$,

3.15 $$\begin{aligned} a_{n}< \pi< b_{n}, \end{aligned}$$

*where*

3.16 $$\begin{aligned} a_{n}=\frac{n^{5}+\frac{5}{4}n^{4}+\frac{51}{16}n^{3}+\frac{133}{64}n^{2}+\frac {5{,}243}{4{,}096}n+\frac{3{,}867}{16{,}384}}{n^{6}+\frac{3}{2}n^{5}+\frac {113}{32}n^{4}+\frac{93}{32}n^{3}+\frac{7{,}729}{4{,}096}n^{2}+\frac {4{,}881}{8{,}192}n+\frac{10{,}395}{131{,}072}} \biggl(\frac{(2n)!!}{(2n-1)!!} \biggr)^{2} \end{aligned}$$

*and*

3.17 $$\begin{aligned} b_{n}=\frac{n^{4}+n^{3}+\frac{107}{64}n^{2}+\frac{91}{128}n+\frac {789}{4{,}096}}{n^{5}+\frac{5}{4}n^{4}+\frac{125}{64}n^{3}+\frac {295}{256}n^{2}+\frac{1{,}689}{4{,}096}n+\frac{945}{16{,}384}} \biggl(\frac {(2n)!!}{(2n-1)!!} \biggr)^{2}. \end{aligned}$$

### Proof

Noting that (3.5) holds, we see by (3.13) that the left-hand side of (3.15) holds for $n\geq4$, while the right-hand side of (3.15) is valid for $n\geq3$. Elementary calculations show that the left-hand side of (3.15) is also valid for $n =1, 2$ and 3, and the right-hand side of (3.15) is valid for $n =1$ and 2. The proof is complete. □

## Comparison

Recently, Lin [12] improved Mortici’s result (1.3) and obtained the following inequalities:

4.1 $$ \lambda_{n}< \pi< \mu_{n} $$

and

4.2 $$ \delta_{n}< \pi< \omega_{n}, $$

where

4.3 $$\begin{aligned} & \lambda_{n}= \biggl(1+ \frac{1}{4n}-\frac{3}{32n^{2}}+\frac{3}{128n^{3}}+\frac {3}{2{,}048n^{4}}- \frac{33}{8{,}192n^{5}}-\frac{39}{65{,}536n^{6}} \biggr) \\ &\phantom{\lambda_{n}=}{}\times \frac {2}{2n+1} \biggl( \frac{(2n)!!}{(2n-1)!!} \biggr)^{2}, \end{aligned}$$

4.4 $$\begin{aligned} & \mu_{n}= \biggl(1+ \frac{1}{4n}-\frac{3}{32n^{2}}+\frac{3}{128n^{3}}+\frac {3}{2{,}048n^{4}} \biggr)\frac{2}{2n+1} \biggl(\frac{(2n)!!}{(2n-1)!!} \biggr)^{2}, \end{aligned}$$

4.5 $$\begin{aligned} &\delta_{n}= \biggl( \frac{(2n)!!}{(2n-1)!!} \biggr)^{2} \frac{1}{n}\exp \biggl(- \frac{1}{4n}+\frac{1}{96n^{3}}-\frac {1}{320n^{5}}+\frac{17}{7{,}168n^{7}}- \frac{31}{9{,}216n^{9}} \biggr), \end{aligned}$$

4.6 $$\begin{aligned} & \omega_{n}= \biggl( \frac{(2n)!!}{(2n-1)!!} \biggr)^{2} \frac{1}{n}\exp \biggl(- \frac{1}{4n}+\frac{1}{96n^{3}}-\frac {1}{320n^{5}}+\frac{17}{7{,}168n^{7}} \biggr). \end{aligned}$$

Direct computation yields

$$\begin{aligned} &a_{n}-\lambda_{n} \\ &\quad=\frac {3(7{,}634{,}944n^{5}+12{,}928{,}000n^{4}+18{,}895{,}616n^{3}+9{,}755{,}072n^{2}+1{,}930{,}008n+135{,}135)}{32{,}768n^{6}(2n+1)(131{,}072n^{6} +196{,}608n^{5}+462{,}848n^{4}+380{,}928n^{3}+247{,}328n^{2}+78{,}096n+10{,}395)} \\ &\qquad{} \times \biggl(\frac{(2n)!!}{(2n-1)!!} \biggr)^{2}>0 \end{aligned}$$

and

$$\begin{aligned} &b_{n}-\mu_{n} \\ &\quad =-\frac {3(45{,}056n^{4}+62{,}976n^{3}+66{,}496n^{2}+21{,}876n+945)}{1{,}024n^{4}(2n+1) (16{,}384n^{5}+20{,}480n^{4}+32{,}000n^{3}+18{,}880n^{2}+6{,}756n+945)} \biggl(\frac{(2n)!!}{(2n-1)!!} \biggr)^{2}\\ &\quad < 0. \end{aligned}$$

Hence, (3.15) improves (4.1).

The following numerical computations (see Table 1) would show that $\delta_{n}< a_{n}$ and $b_{n}<\omega_{n}$ for $n\in\mathbb{N}$. That is to say, inequalities (3.15) are sharper than inequalities (4.2).

**Table 1:** **Comparison between inequalities** (**3.15**) **and** (**4.2**)

***n***	$\boldsymbol {a_{n}-\delta_{n}}$	$\boldsymbol {\omega_{n}-b_{n}}$
1	6.673798 × 10^−3^	3.789512 × 10^−3^
10	2.264856 × 10^−13^	9.947434 × 10^−12^
100	2.398663 × 10^−24^	1.051407 × 10^−20^
1,000	2.408054 × 10^−35^	1.056218 × 10^−29^
10,000	2.408948 × 10^−46^	1.056690 × 10^−38^

In fact, we have

$$\begin{aligned} &\lambda_{n}= \pi+O \biggl(\frac{1}{n^{7}} \biggr),\qquad \mu_{n}=\pi+O \biggl(\frac {1}{n^{5}} \biggr), \\ & \delta_{n}=\pi+O \biggl(\frac{1}{n^{11}} \biggr),\qquad \omega_{n}=\pi+O \biggl(\frac{1}{n^{9}} \biggr), \\ &a_{n}= \pi+O \biggl(\frac{1}{n^{12}} \biggr), \qquad b_{n}= \pi+O \biggl(\frac {1}{n^{10}} \biggr). \end{aligned}$$
